# Circuits Regulating Superoxide and Nitric Oxide Production and Neutralization in Different Cell Types: Expression of Participating Genes and Changes Induced by Ionizing Radiation

**DOI:** 10.3390/antiox9080701

**Published:** 2020-08-03

**Authors:** Patryk Bil, Sylwia Ciesielska, Roman Jaksik, Joanna Rzeszowska-Wolny

**Affiliations:** 1 Department of Systems Biology and Engineering, Faculty of Automatic Control, Electronics and Computer Science, Silesian University of Technology, 44-100 Gliwice, Poland; Patryk.Bil@polsl.pl (P.B.); Sylwia.Ciesielska@polsl.pl (S.C.); Roman.Jaksik@polsl.pl (R.J.); 2 Biotechnology Centre, Silesian University of Technology, 44-100 Gliwice, Poland

**Keywords:** superoxide radical, nitric oxide, nitric oxide synthase, ionizing radiation

## Abstract

Superoxide radicals, together with nitric oxide (NO), determine the oxidative status of cells, which use different pathways to control their levels in response to stressing conditions. Using gene expression data available in the Cancer Cell Line Encyclopedia and microarray results, we compared the expression of genes engaged in pathways controlling reactive oxygen species and NO production, neutralization, and changes in response to the exposure of cells to ionizing radiation (IR) in human cancer cell lines originating from different tissues. The expression of NADPH oxidases and NO synthases that participate in superoxide radical and NO production was low in all cell types. Superoxide dismutase, glutathione peroxidase, thioredoxin, and peroxiredoxins participating in radical neutralization showed high expression in nearly all cell types. Some enzymes that may indirectly influence superoxide radical and NO levels showed tissue-specific expression and differences in response to IR. Using fluorescence microscopy and specific dyes, we followed the levels and the distribution of superoxide and NO radicals in living melanoma cells at different times after exposure to IR. Directly after irradiation, we observed an increase of superoxide radicals and NO coexistent in the same subcellular locations, suggesting a switch of NO synthase to the production of superoxide radicals.

## 1. Introduction

Reactive oxygen species (ROS) appear in different metabolic reactions in organisms living in aerobic conditions. The main representatives of ROS are superoxide radical (O_2_^•^^−^), hydroxyl (^•^OH), singlet oxygen (^1^O_2_), and hydrogen peroxide (H_2_O_2_), which together with reactive nitrogen species such as nitric oxide (NO), peroxynitrite (ONOO^−^), and nitrogen dioxide (NO_2_) are damaging agents causing death and involved in aging; however, they are also important players in cellular regulation mechanisms and signaling pathways [1,2,3,4]. ROS may origin from endogenous or exogenous sources; endogenous sources include mitochondria, peroxisomes, and endoplasmic reticulum, where oxygen consumption is high [5]. Exogenous sources of ROS include ionizing and non-ionizing radiation, drugs, pollutants, food, ultrasound, xenobiotics, and toxins [6]. Low doses of radiation generate small amounts of ROS, causing limited disturbances to the redox state, but higher doses cause chronic oxidative stress, which can constantly disturb redox homeostasis [7]. Most certainly, cells cannot distinguish if ROS are produced by radiation or endogenously by mitochondrial respiration [8]. The dual role of ROS as a damaging or signaling factor depends on their levels. To cope with the permanent presence of oxygen and too high production of oxygen-derived radicals, living organisms have developed cellular systems that equilibrate ROS levels and counteract oxidative stress. Redox homeostasis is maintained by special factors termed antioxidants, which can convert and neutralize ROS by various pathways. Antioxidants are chemical species or enzymes that can break the chains of cellular oxidative reactions in which free radicals appear. They can be produced by cells or, similar to some vitamins, taken from the environment. Among the enzymes participating in the maintenance of proper cellular redox conditions are superoxide dismutase (SOD), which converts superoxide radical to H_2_O_2_ [9]; subsequently, H_2_O_2_ can be further converted to H_2_O by catalase (CAT), peroxiredoxins (PRDX), or glutathione peroxidase (GPX). Superoxide radicals can also react with NO to form ONOO^−^ [10,11,12,13,14,15,16]

On the other hand, cells contain special enzymes that can directly and purposely produce superoxide radical and NO, such as nitric oxide synthases (NOS) and NADPH oxidases (NOX), and this production is also important for the maintenance of redox homeostasis and participation of ROS in cellular signaling pathways. Both NOX and NOS families of enzymes are important regulators of cell differentiation, growth, proliferation, and they are also mechanisms that are important for a wide range of processes from embryonic development through tissue regeneration to the development and spread of cancer. Redox homeostasis is important for all these processes, and it depends on the production and neutralization of ROS, which engages not only the above-mentioned enzymes but further sometimes complicated pathways, providing substrates for particular reactions [6,17]. 

In the present work, we compared the expression levels of genes coding for enzymes producing ROS and NO, as well as others participating in the neutralization of ROS, in more than 1000 human cell lines originating from different tissues and cancers in the Cancer Cell Line Encyclopedia database [18] and our microarray data. The levels of most of these enzymes are similar in different cell types, showing low expression of those producing superoxide radicals but high expression of those neutralizing superoxide radicals; however, some tissue specificity was seen. The changes of expression of genes engaged in redox homeostasis were examined in three cell types exposed to ionizing radiation, and the results suggest that different cell types use different redox pathways to cope with redox stress. 

## 2. Materials and Methods 

### 2.1. Analysis of the Levels of Transcripts Coding for Proteins Engaged in Cellular Redox Processes in HCT116, K562, and Me45 Cells

To study the changes in transcriptomes induced by irradiation, we used HCT116 (a human colon carcinoma cell line established from the primary colon carcinoma of an adult man, ATCC-CCL247), K562 (an erythroleukemia cell line derived from a chronic myeloid leukemia patient in blast crisis, ATCC-CCL243), and Me45 (obtained from Dr M. Widel who established this cell line in the Center of Oncology in Gliwice from a lymph node metastasis of skin melanoma in 1997). Cells were exposed to 4Gy of ionizing radiation (1 Gy/min from a Clinac 600 GMV system), and transcriptome changes were assayed using Affymetrix microarray methods. The culture and irradiation conditions together with the microarray methods were described earlier [19,20], and the results are available in the ArrayExpress database under accession number E-MEXP-2623 [21]. All data are Minimum Information About a Microarray Experiment (MIAME) compliant. Microarray data quality was assessed using the simpleaffy Bioconductor package [22]. Raw HG-U133A microarray data obtained from two experiments, based on HCT116, K562, and Me45 cell lines, were processed using the Brainarray EntrezGene specific custom CDF (v22) [23] in R using the Robust MultiArray Average (RMA) algorithm implemented in the Affy Bioconductor library [24]. Differentially expressed genes were identified using limma with q-value correction for multiple testing [25,26].

Genes coding for proteins engaged in cellular redox processes were identified using Gene Ontology (GO) terms [27,28] such as oxide, superoxide, nitric oxide, hydrogen peroxide, ROS, and reactive oxygen species, NO synthase, and NADPH oxidases. The levels of transcripts of these genes in non-irradiated and irradiated cells were extracted from the ArrayExpress database. 

### 2.2. Expression of Redox Engaged Genes in Different Cell Types 

To compare the expressions of genes coding for proteins active in redox processes, we extracted RNA-seq data from the Cancer Cell Line Encyclopedia [18] that originate from 1025 different cell lines derived from various tissues. Standardized read counts were compared on a logarithmic scale between 41 genes grouped into 6 categories based on their involvement in redox processes; then, they were visualized as a heatmap. We additionally focused on a group of nine specific cell lines for which we visualized the expression levels using individual markers, providing a mean and standard deviation for each of the genes.

### 2.3. Microscopic Observation and Analysis

The levels of nitric oxide and superoxide and their changes were analyzed in living cells using an Olympus CellR fluorescence microscope with a chamber for live observation and dedicated OV100 software. Cells were seeded in two dishes (35 mm diameter) (5000/dish) in colorless DMEM/F12 medium (PAN Biotech, Aidenbach, Germany, cat. no. P04-41650) enriched with 10% fetal bovine serum (EURx, Gdansk, Poland cat# E5050-03-500), incubated in standard conditions for 48 h, and then MitoSOX Red (Thermo Fisher Scientific, Waltham, USA, cat. no. M36008) and DAF-FM diacetate (Thermo Fisher Scientific, Waltham, USA, cat.# D23844) both at a final concentration of 5 µM were added to the medium. After 1 h of incubation, the medium containing dyes was replaced by fresh medium, the cells on one dish were irradiated with ionizing radiation (4Gy), and both dishes were placed in a chamber for live observation at 37 °C in 5% CO^2^. Images were acquired in three channels: red fluorescence, green fluorescence (wavelengths adequate for used fluorescent dyes), and transparency, every 0.5 h for 48 h. 

Computational analysis of images was performed with ImageJ software (1.48f version) [29] for the manual segmentation of cells and creation of binary masks. Matlab 2019b (MathWorks, Natick, Massachusetts, USA) was used for calculating Pearson’s correlation [30] between the brightness in the same pixels on two images taken from the same microscopic field but using different filters for MitoSOX Red and DAF-FM. The correlation was calculated to estimate the level of co-localization of two types of radical.

### 2.4. Construction of the Novel Interaction Schemes

Superoxide radical production and neutralization schemes were based on data from publications [1,9,17,31] and data for reactions of H_2_O_2_ neutralization from [12,13,14,15,16,32,33]. The data on NOS-based production of O_2_^•^^−^ and NO were found in [34,35,36,37]. Data for the urea cycle providing L-arginine, the NOS substrate, were obtained from [38,39,40], and those for the production and regeneration of tetrahydrobiopterin were from [41,42].

## 3. Results

### 3.1. Pathways of Cellular Superoxide and NO Production and Neutralization 

**Figure 1:**
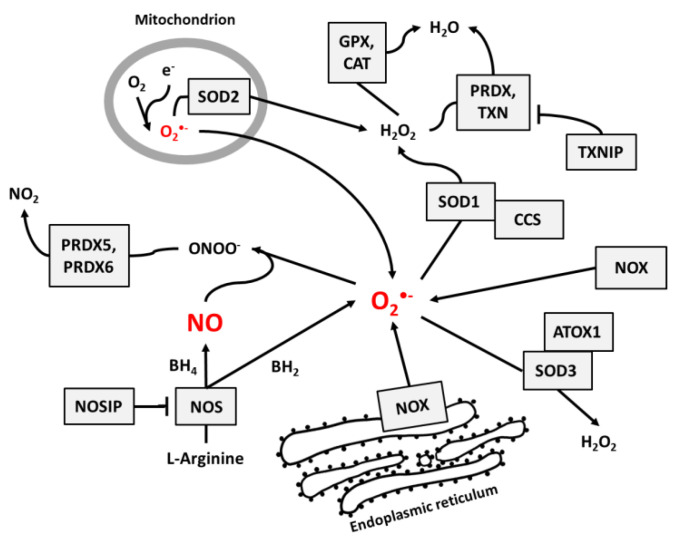
The main pathways responsible for the regulation of NO and superoxide radical levels in living cells. Rectangles indicate participating proteins and arrows indicate the direction and products of reactions.

**Figure 2:**
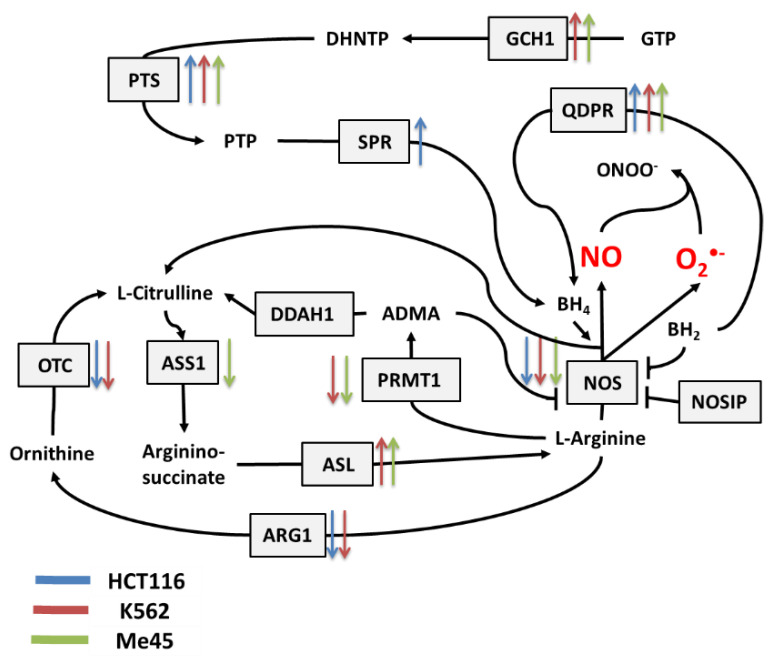
Cellular pathways influencing the production and neutralization of NO. The colored arrows show the direction of change (up/down regulation) of enzyme mRNA levels after the exposure of melanoma Me45 (green), K562 (red), and HCT116 (blue) cells to ionizing radiation.

**Figure 3:**
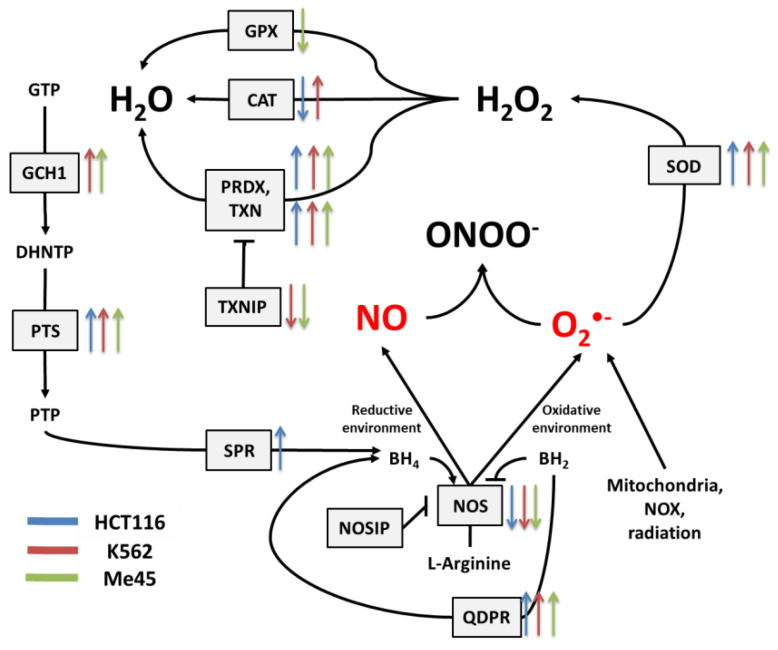
The tetrahydrobiopterin (BH4) pathway in NO and superoxide radical production and neutralization. The colored arrows show the direction of change of enzyme mRNA levels after the exposure of melanoma Me45 (green), K562 (red), and HCT116 (blue) cells to ionizing radiation.

NO and superoxide are crucial radicals responsible for the redox status of each cell. They seem to be the only ROS that are produced purposely in all cell types and whose levels are precisely controlled by different regulatory circuits. Collecting different data from the literature, we created schemes of reactions interconnecting different enzymes and their products, which directly or indirectly participate in the regulation of superoxide and NO levels in cells (Figure 1, Figure 2 and Figure 3). 

Figure 1 presents the main pathways by which superoxide and NO may be produced and neutralized in most cells. Among the wide range of ROS, O_2_^•^^−^ is thought to be the primary radical present in eukaryotic cells living in an aerobic environment [43]. It is created when oxygen accepts an additional electron [1], which is a reaction that may be catalyzed by NADPH oxidases [17], NO synthases [34,35] (due to normal metabolism with electron leakage in mitochondria), peroxisomes, nuclear membranes, and cytoplasm [1], or by radiation [31]. Superoxide radicals are neutralized by SOD enzymes, which catalyze the conversion of O_2_^•^^−^ to H_2_O_2_. In mitochondria, O_2_^•^^−^ neutralization is performed by Mn-dependent SOD2, and in cytosol and extracellular space, it is performed by Cu- and Zn-dependent SOD1 and SOD3 [9]. At higher concentrations, O_2_^•^^−^ molecules can also convert to water spontaneously [1]. 

Catalase (CAT) [12], peroxiredoxins (PRDX) [13,14], and glutathione peroxidases (GPX) [15,16] are responsible for H_2_O_2_ neutralization to water. Peroxiredoxines (also called thioredoxin peroxidases) are thiol-specific antioxidant enzymes. They serve as antioxidants to H_2_O_2_, ONOO^−^, and other peroxides, and they can reduce even more than 90% of cellular peroxides. Peroxiredoxins are widely spread and in some organisms may constitute up to 1% of total proteins [13]. PRDXs 1 to 3 and PRDX 5 and 6 are localized in the cytosol, mitochondria, nuclei, and peroxisomes, and PRDX4 is localized in the endoplasmic reticulum, from where it can be secreted to the extracellular space [32,33]. 

GPX action is based on glutathione (GSH), one of the most important cellular protectors from toxic ROS effects, which is widely produced in all cells and contains more than 90% of total non-protein sulfur. The system for GSH turnover between reduced and oxidized forms performed by glutathione reductases and peroxidases is very important for cellular redox homeostasis [15].

Figure 2 shows the main pathways influencing the production and neutralization of NO, which plays important roles in intercellular and intracellular signaling and is produced by nitric oxide synthases (NOS1, NOS2, and NOS3). All NOS enzymes work as dimers and contain reductase and oxygenase domains in their structure. They produce NO in a reaction where L-arginine as substrate is converted to L-citrulline and NO [36,37]. One of the most interesting characteristics of NOS is its ability to switch between the production of NO and superoxide radical [44]. The switch mechanism is based on the availability of tetrahydrobiopterin (BH4) and its oxidized form, dihydrobiopterin (BH2) (Figure 2 and Figure 3). Tetrahydrobiopterin is a cofactor of NOS that is necessary for the conversion of L-arginine into L-citrulline and nitric oxide [36]. Dihydrobiopterin competes with BH4 for binding sites on NOS, and when bound to the enzyme, it changes NOS functionality in such a way that superoxide radical is produced instead of L-citrulline and NO [45]. In an environment with a low risk of cellular stress, the NOS activity is directed toward NO generation. Stressing factors that change oxidative conditions may promote the oxidation of BH4 to BH2, and this will upregulate superoxide radical production by NOS [45]. The activity of NOS can be also regulated by the nitric oxide synthase interacting protein (NOSIP) [46]. 

The bioavailability of L-arginine, a substrate for NO production, is related to activity of the urea cycle in which the following enzymes participate: protein arginine N-methyltransferase 1 (PRMT1), dimethylargininase 1 (DDAH1), argininosuccinate synthase 1 (ASS1), argininosuccinate lyase (ASL), arginase 1 (ARG1), and ornithine transcarbamylase (OTC) (Figure 2). 

In the urea cycle, the conversion of L-arginine to L-citrulline can go through two pathways: the first involves PRMT1 and DDAH1, and the second relies on ARG1 and OTC. The intermediates in these processes are asymmetric dimethylarginine (ADMA) and ornithine, respectively. Citrulline produced in both pathways serves for L-arginine regeneration, which is carried by ASS1 and ASL—enzymes with arginino-succinate as intermediate. Keeping the proper arginine level is important for the production of proteins, but it is also crucial for NO synthesis; however, the level of NO must be under strict control, as this radical participates in different signaling pathways [38,39,40]. One of the possible regulatory mechanisms is the inhibition of NOS by switching its activity from the production of NO to the production of superoxide radical based on the oxidation of BH4.

The cellular level of BH4 depends on a pathway including GTP cyclohydrolase 1 (GCH1), pyruvoyl tetrapterin synthase (PTS), and sepiapterin reductase (SPR). This path is responsible for an increase in unoxidized biopterin levels: GCH1 converts GTP into dihydroneopterin triphosphate (DHNTP), and then PTS uses this to produce pyruvoyl tetrapterin (PTP), and finally, SPR processes pyruvoyl tetrapterin into tetrahydrobiopterin (Figure 2 and Figure 3). BH4 can be oxidized to BH2 by superoxide radical, and other elements that can counteract this effect are superoxide dismutases (SODs), which can limit superoxide radical levels [9]. The product of SOD action is H_2_O_2_, which positively influences the production of GTP cyclohydrolase 1 in some cells [41]. BH4 can be also regenerated from BH2 by dihydropteridine reductase (QDPR), thus helping cells to keep redox balance [42].

### 3.2. Expression of Transcripts for Enzymes Participating in Redox Regulation in Different Cell Types

**Figure 4:**
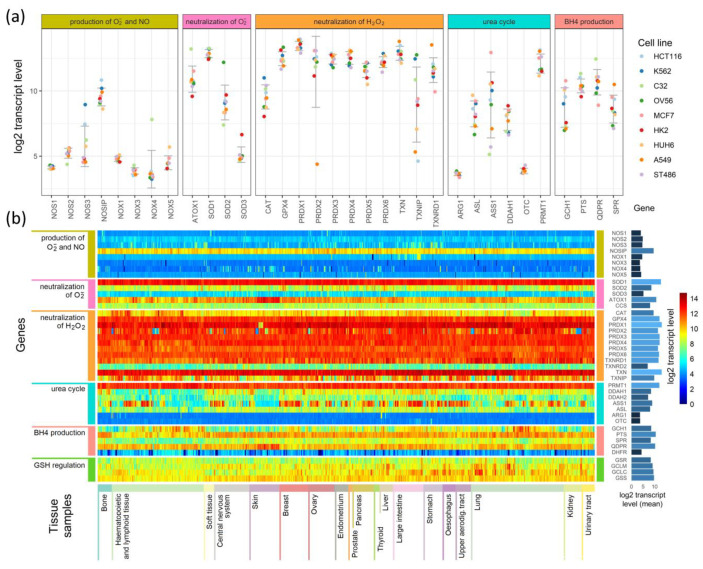
Comparison of the expression of enzymes participating in the production of superoxide radicals and NO and their neutralization in (**a**) 9 cell lines and (**b**) a heatmap comparison of such expression in 1025 cell lines derived from different tissues.

To search for relationships between different pathways engaged in redox processes in different cell types, we investigated the expression of genes shown in Figure 1, Figure 2 and Figure 3. The expression levels of these genes in 1025 cell lines were obtained from the Cancer Cell Line Encyclopedia [18]. The results of this analysis are presented in Figure 4.

Figure 4a presents expression levels in nine selected cell lines, where genes were grouped by their involvement in the processes shown in Figure 1, Figure 2 and Figure 3, and colored points show the levels of transcripts in particular cell lines. As examples, we chose cell lines of common neoplasms derived from various tissues. In Figure 4b, we present the results for all 1025 cell types as a heatmap. The cells (in columns) were grouped according to the tissue from which they have been derived, and the genes (in rows) were grouped similarly as in Figure 4a. On the right side of the heatmap, we give the mean values of each gene transcript levels (log2) in all cell lines. 

The expression of genes engaged in the production and neutralization of O_2_^•^^−^ and NO are similar in most of the cells, but some cell types and tissue specificity are also seen (Figure 4a,b). Genes coding for proteins directly participating in O_2_^•^^−^ and NO production have low levels of transcripts in all cell types. The main enzymes that direct the neutralization of hydrogen peroxide show mostly conserved expression among different cell lines, but in contrast, they are characterized by relatively high expression (Figure 4b). However, superoxide dismutases, which neutralize O_2_^•^^−^, have an expression that strictly depends on the isoenzyme: SOD1 has the highest, SOD3 has the lowest, and SOD2 has an expression that is halfway between the other two (Figure 4a,b). ATOX1, which donates copper ions to SOD3, seems to have a cell line-dependent expression that is especially high in skin cancer cell lines (it is unclear if it is skin cancer cell-specific or also observed in normal skin cells) (Figure 4a,b). The genes that are responsible for the production and regeneration of BH4, and those that are part of the urea cycle, have cell-line specific expression that in some cases seems to be related to the tissue of origin and in others is strictly related to a single cell line (Figure 4b). 

Further examination shows that some genes have tissue-based expression. In comparison to other tissues, bone cells have generally higher PTS and TXNRD1 expression, and a lower expression of GCH1. Hematopoietic and lymphoid tissue are characterized by lower transcript levels for SPR, DDAH1, and DDAH2. However, many cells of these tissues have a higher than average expression of CAT gene transcripts. The most distinguishing feature of central nervous system and kidney cells are an overall lower expression of GCH1 (BH4 production) compared to other tissues. Cells of the upper aerodigestive tract have on average a lower expression of DDAH2 than other cell types. Among the tissues analyzed, skin cells (next to hematopoietic and lymphoid cells) are the most distinctive in their expression of selected genes: they have a higher than average expression of QDPR and ATOX1 genes, and a visibly lower expression of GCH1, DDAH2, and ASS1. Other tissues have expression levels that do not differ significantly from average, but some are not represented by a sufficient number of cell lines to identify tissue-specific differences in expression.

### 3.3. Irradiation-Induced Changes of Expression of Enzymes Responsible for Superoxide and NO Radicals Production

**Figure 5:**
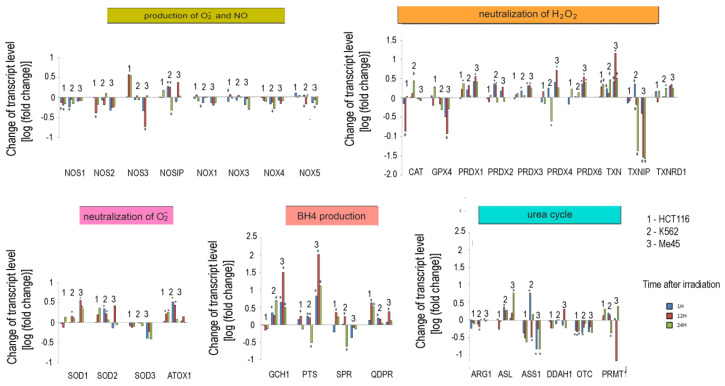
Response to ionizing radiation exposure in three cell lines: HCT116 (1), K562 (2), and Me45 (3) assayed at 1 h, 12 h, and 24 h after irradiation with 4 Gy of gamma rays (blue, red, and green bars, respectively). To better show the direction of changes, the results are presented as log2 of the fold change. Asterisks mark statistically significant fold changes.

Our analyses of the levels of reactive oxygen and nitrogen species in cells exposed to UV or ionizing radiation showed that these treatments may significantly change the redox conditions and proliferation of cells [19,47]. Thus, in further work, we analyzed the ionizing radiation-induced changes in the expression of genes related to the pathways responsible for the regulation of O_2_^•^^−^ and NO levels in three cell lines originating from different tissues. HCT116, K562, and Me45 cells were exposed to ionizing radiation at a dose of 4Gy and incubated for 24 h in standard conditions. Transcriptomes of samples taken at 1, 12, and 24 h were assayed by microarray methods. The changes of the levels of transcripts are presented in Figure 5. 

In general, the expression of genes coding for enzymes participating in the production of superoxide radical and NO decreased in cells exposed to ionizing radiation; the decreases were statistically significant but not considerable. Ionizing radiation caused a general increase in the levels of transcripts participating in radical neutralization, and we also observed a significant decrease in thioredoxin interacting protein (TXNIP), the inhibitor of thioredoxin (TXN), in all cell lines. The expression of PRDX and TXN genes increased after irradiation in all cell types, but cell line-specific changes were seen in CAT and GPX gene expression, suggesting that they may differently manage the neutralization of hydrogen peroxide.

Irradiation creates an oxidative environment in cells, and any excess superoxide radical is most likely processed into hydrogen peroxide by SODs. After irradiation, the expression of SOD1 and SOD2 genes increased in Me45, HCT116, and K562 cells to a similar extent. Interestingly, SOD3 transcripts showed a decrease after irradiation in all three cell types. 

Radiation also caused a significant rise in the expression of genes whose products participate in BH4 production, although in contrast, the expression of genes participating in the urea cycle decreased. BH4 production seems to be an important element of the response to radiation. In K562 and Me45 cells, the expression of GCH1 and PTS genes, which are responsible for the production of BH4 from GTP, increased significantly after irradiation. The change of GCH1 transcript level was much smaller in HCT116 cells, but these cells show a higher level of GCH1 transcription in non-irradiated cells. All three cells types exposed to radiation showed a raised expression of the QDPR gene, and HCT116 had a raised expression of the SPR gene, which is also part of the BH4 production path. The reason for these changes in the expression seems to be the need for the regeneration of the decreasing pool of BH4 necessary for NO production by NOS in an oxidative environment. 

The changes in expression after irradiation in all the cell lines studied (HCT116, K562, and Me45) appear to switch the urea cycle toward limiting citrulline production and the regeneration of arginine, which is plausibly to support the production of NO by NOS [Figure 2]. Me45 cells suppress PRMT1 expression to reduce the conversion of arginine to citrulline, while HCT116 cells achieve a similar effect by suppressing OTC and ARG1 genes. Additionally, Me45 cells had increased levels of ASL transcripts to support the regeneration of arginine. Similar effects of radiation on the urea cycle genes in Me45 and HCT116 were also observed in the K562 genes, suggesting that these cells use all of these mechanisms to focus on arginine production and conservation.

### 3.4. Changes of NOS Activity in Response to Ionizing Radiation

**Figure 6:**
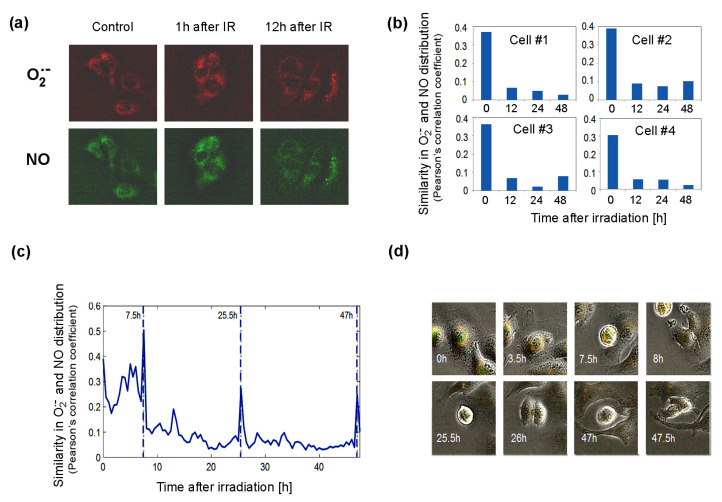
Distribution of superoxide and NO radicals in living control and 4Gy-irradiated (IR) Me45 cells. (**a**) Fluorescence microscopy detection of O_2_^•^^−^ and NO labeled respectively with MitoSOX Red and DAF-FM diacetate. (**b**) Correlation between O_2_^•^^−^ and NO distributions (measured by Pearson’s correlation coefficient) in four irradiated cells. (**c**) Changes of correlation between O_2_^•^^−^ and NO concentrations (measured by Pearson’s correlation coefficient) during cell cycle in a single irradiated Me45 cell, with marked cell division time points. (**d**) Images of the cell shown in (**c**) at selected time points.

The significant changes after irradiation in the expression of genes in pathways governing the production of BH4, a factor important for NOS activity, suggest that NOS uncoupling may be important for cellular responses to radiation. To approach this problem, we used time-lapse fluorescent microscopy to study living Me45 cells, which were exposed to 4Gy of gamma radiation and observed over 48 h. Cells were labeled with the fluorescent probes MitoSOX Red, which interacts specifically with the superoxide radical, and DAF-FM, which detects NO. These observations showed that both these radicals form foci that often are unevenly distributed in cells (Figure 6a). 

The superoxide radical was detected in the cytoplasm in most cells, but occasionally it was also in the nucleus. We followed the distribution of both radicals, collecting fluorescence data for each pixel in a single cell image at 0.5 h time intervals over 48 h. The distributions of O_2_^•^^−^ and NO were compared by the calculation of correlations of the concentrations in all pixels of the cell for each time point. As superoxide radicals have a very short lifetime, high correlation coefficients should reflect a very close distance between the sources of both radicals. The results showed that shortly after irradiation, the correlation is high, suggesting that both radicals and their sources are present in the same areas of the cell (Figure 6b). The correlation diminished with time, which suggests that the NO and O_2_^•^^−^ sources were separating spatially from each other (Figure 6b). The appearance of superoxide radicals and NO in the same places in the cell after irradiation, which was reflected by an increase in the correlation coefficient, could result from the uncoupling of NOS activity (decrease in BH4, whose oxidation may be induced by ionizing radiation) and increase in the production of O_2_^•^^−^ by these enzymes. The irradiation-induced significant overlapping of O_2_^•^^−^ and NO distributions (Pearson’s correlation coefficient of about 0.4) did not persist long and decreased a few hours after irradiation (Figure 6b). In spite of the exposure to ionizing radiation, the group of cells observed proliferated quite frequently. An increase in the correlation of the distribution of superoxide radical and NO was also observed during cell division, and spikes occurred in Pearson’s correlation between NO and O_2_^•^^−^ localization just before cell division (Figure 6c).

## 4. Discussion

Here, we show the results of analyses of the expression of genes coding for enzymes that participate in the regulation of cellular redox status, comparing the levels of transcripts for these genes in more than 1000 cell types from different tissues, including cancer cell lines. We used the unique resources of the Cancer Cell Line Encyclopedia database [18] to obtain data for thousands of cell lines that can be directly compared. 

The main striking feature of our analysis is the marked intercellular and inter-tissue similarity of transcript profiles for genes coding enzymes engaged in the production of NO and O_2_^•^^−^ (low expression) or in the neutralization of H_2_O_2_ (high expression), which shows that some of the mechanisms that regulate radical levels must be common to many cell types. High and very similar transcript levels were observed for PRDX, TXN, and TXNRD1 in all cells. In the case of PRDX expression, this similarity as well as the change in response to radiation is not surprising, because these proteins are highly conserved and play similar roles in different tissues and organisms [48]. Peroxiredoxines participate in the cellular signaling of reactive oxygen species, and they may play a role as chaperones and also participate in non-canonical transcript-independent circadian clocks [48]. They exist as dimers that can form intramolecular disulfide bridges that are reduced by thioredoxin (TRX), which is in turn reduced by thioredoxin reductases utilizing NADPH as a source of reducing power [49]. Thus, PRDX together with TXN and TXNRD1 comprise a regulatory circuit, and one could expect that all its elements will be expressed at a similar level in all cell types. TXN is overexpressed in many human cancers, and high levels appear to be related to decreased patient survival [50,51], while increased transcript levels have been found in primary human lung and colorectal cancers [52]. Moreover, studies of Hoshi 1997 revealed that IR induces increased levels of not only transcripts but also proteins in human lymphocytes [53]. Tumors with higher TXN have increased cell growth and inhibited apoptosis [51,52]. The overexpression of TXN in lung carcinomas and the subsequent redox regulation and activation of a number of intracellular proteins that control cell growth and proliferation may be an indication of more aggressive tumor phenotypes [54]. Altogether, these features show that TXN is an important regulator of redox homeostasis and seems to be a crucial element of the PRDX–TXN circuit.

Further genes with high and similar expression in all tissues are SOD1, which converts superoxide radicals to H_2_O_2_, and GPX4, which neutralizes H_2_O_2_. These enzymes, but not catalase, may belong to the same ancient conserved system as PRDX and TXN, which regulates redox homeostasis and also displays an endogenous circadian rhythm that is interconnected with circadian clocks based on gene expression feedback loops [48,49]. Catalase, which shows lower and tissue-specific expression (Figure 5), is most probably is not an element of the same pathway. Another gene expressed equally highly in all cell types is that coding for protein arginine methyltransferase 1, which is regulated by oxidation and produces ADMA, which is a NOS inhibitor [55]. The high level of PRMT1 in all cell lines suggests that the regulation of NO levels is crucial for cellular homeostasis in all cell types. NO levels are regulated by the modulation of NOS activity, which depends on the availability of cofactors and the activity of enzymes that produce and regenerate them. The main NOS cofactor is BH4, whose production goes through a series of reactions carried out by the enzymes GCH1, PTS, and QDPR. In HCT116, K562 and Me45 cells’ exposure to radiation induces an increased expression of these genes (Figure 5). BH4 seems to be especially important in the cellular oxidative stress response, as it is needed for NO production from L-arginine, and in oxidative conditions, BH2 appears, which uncouples NOS and switches the enzyme to superoxide radical production [34]. Here, we propose a new method for estimating this NOS uncoupling by measuring the correlation between the intracellular distribution of NO and O_2_^•^^−^ detected by specific fluorescent probes. We observed that the sources of these two radicals co-localize and that this correlation increases in oxidative conditions created by exposure to ionizing radiation. We hypothesize that this effect most probably results from NOS uncoupling; radiation induces an oxidative environment, resulting in the increased oxidation of BH4 to BH2 and causing a switch from NO production to superoxide radical production. This co-localization decreases over time (Figure 6), which is probably due to cells regaining their balanced redox state rise and BH4 to BH2 ratio. The switch of nitric oxide synthases to the production of superoxide radicals can be part of a mechanism that, in response to ionizing radiation, raises the superoxide radical level to a threshold at which further pathways participating in the neutralization of O_2_^•^^−^ are activated.

NO is a signaling molecule that may act as an autocrine or paracrine messenger, which in human organisms regulates many functions, such as blood pressure, neurotransmission, immune response, and oxidation-sensitive mechanisms [56]. The major reactions involving NO include its rapid oxidation and peroxynitrites, which are generated from reaction with a superoxide, can interact with several cellular components, and are implicated in NO signaling mechanisms involving protein modifications [34]. It is possible that for the fast production of specific products of NO and O_2_^•^^−^ interactions, cells use the switch mechanism with NOS uncoupling to have both substrates in one place.

In a time-lapse experiment, we observed sharp increases in the correlation of the distribution of superoxide radicals that appeared just before cell division. This increased overlapping of both radicals in space could be due to the detachment and change of the shape of cells, but one could also speculate that the change of shape that causes an increase in the concentration of reactive oxygen species may have a similar effect as irradiation and cause the switching of NOS for the production both NO and O_2_^•^^−^ radicals in short time intervals, which would speed up the oxidation of NO necessary to create specific signaling molecules for the next steps in cell division. 

Cells have developed systems to maintain ROS levels as stable and counteract oxidative stress. Different cell types use different redox pathways to cope with redox stress. It seems that cells have special mechanisms that are triggered by high doses of radiation. Identifying the mechanisms by which cancer cells adjust to and survive in an oxidative environment may be important in radiotherapy. 

## References

[1] Sarsour E.H., Kumar M.G., Chaudhuri L., Kalen A.L., Goswami P.C. (2009). Redox control of the cell cycle in health and disease. Antioxid. Redox Signal..

[2] Ozcan A., Ogun M. (2015). Biochemistry of Reactive Oxygen and Nitrogen Species. Basic Principles and Clinical Significance of Oxidative Stress.

[3] Thannickal V.J., Fanburg B.L. (2000). Reactive oxygen species in cell signaling. Am. J. Physiol. Lung Cell. Mol. Physiol..

[4] Feinendegen L.E. (1999). The role of adaptive responses following exposure to ionizing radiation. Hum. Exp. Toxicol..

[5] Phaniendra A., Jestadi D.B., Periyasamy L. (2015). Free Radicals: Properties, Sources, Targets, and Their Implication in Various Diseases. Indian J. Clin. Biochem..

[6] Kohen R., Nyska A. (2002). Oxidation of biological systems: Oxidative stress phenomena, antioxidants, redox reactions, and methods for their quantification. Toxicol. Pathol..

[7] Zhang J., Xing D., Gao X. (2008). Low-power laser irradiation activates Src tyrosine kinase through reactive oxygen species-mediated signaling pathway. J. Cell. Physiol..

[8] Feinendegen L.E., Pollycove M., Sondhaus C.A. (2004). Responses to Low Doses of Ionizing Radiation in Biological Systems. Nonlinearity Biol. Toxicol. Med..

[9] Candas D., Li J.J. (2014). MnSOD in oxidative stress response-potential regulation via mitochondrial protein influx. Antioxid. Redox Signal..

[10] Virág L., Szabó E., Gergely P., Szabó C. (2003). Peroxynitrite-induced cytotoxicity: Mechanism and opportunities for intervention. Toxicol. Lett..

[11] Kamat J.P. (2006). Peroxynitrite: A potent oxidizing and nitrating agent. Indian J. Exp. Biol..

[12] McBride A.G., Borutaité V., Brown G.C. (1999). Superoxide dismutase and hydrogen peroxide cause rapid nitric oxide breakdown, peroxynitrite production and subsequent cell death. Biochim. Biophys. Acta.

[13] Immenschuh S., Baumgart-Vogt E. (2005). Peroxiredoxins, oxidative stress, and cell proliferation. Antioxid. Redox Signal..

[14] Poynton R.A., Hampton M.B. (2014). Peroxiredoxins as biomarkers of oxidative stress. Biochim. Biophys. Acta Gen. Subj..

[15] Meister A. (1988). Glutathione metabolism and its selective modification. J. Biol. Chem..

[16] Lu S.C. (2009). Regulation of glutathione synthesis. Mol. Asp. Med..

[17] Skonieczna M., Hejmo T., Poterala-Hejmo A., Cieslar-Pobuda A., Buldak R.J. (2017). NADPH Oxidases: Insights into Selected Functions and Mechanisms of Action in Cancer and Stem Cells. Oxid. Med. Cell. Longev..

[18] Ghandi M., Huang F.W., Jané-Valbuena J., Kryukov G.V., Lo C.C., McDonald E.R., Barretina J., Gelfand E.T., Bielski C.M., Li H. (2019). Next-generation characterization of the Cancer Cell Line Encyclopedia. Nature.

[19] Herok R., Konopacka M., Polanska J., Swierniak A., Rogolinski J., Jaksik R., Hancock R., Rzeszowska-Wolny J. (2010). Bystander Effects Induced by Medium From Irradiated Cells: Similar Transcriptome Responses in Irradiated and Bystander K562 Cells. Int. J. Radiat. Oncol. Biol. Phys..

[20] Rzeszowska-Wolny J., Herok R., Widel M., Hancock R. (2009). X-irradiation and bystander effects induce similar changes of transcript profiles in most functional pathways in human melanoma cells. DNA Repair.

[21] Athar A., Fullgrabe A., George N., Iqbal H., Huerta L., Ali A., Snow C., Fonseca N.A., Petryszak R., Papatheodorou I. (2018). ArrayExpress update—From bulk to single-cell expression data. Nucleic Acids Res..

[22] Wilson C.L., Miller C.J. (2005). Simpleaffy: A BioConductor package for Affymetrix Quality Control and data analysis. Bioinformatics.

[23] Dai M., Wang P., Boyd A.D., Kostov G. (2005). Evolving gene/transcript definitions significantly alter the interpretation of GeneChip data. Nucleic Acids Res..

[24] Gautier L., Cope L., Bolstad B.M., Irizarry R.A. (2004). affy—Analysis of *Affymetrix GeneChip* data at the probe level. Bioinformatics.

[25] Ritchie M.E., Phipson B., Wu D., Hu Y., Law C.W., Shi W., Smyth G.K. (2015). *limma* powers differential expression analyses for RNA-sequencing and microarray studies. Nucleic Acids Res..

[26] Storey J.D., Tibshirani R. (2003). Statistical significance for genomewide studies. Proc. Natl. Acad. Sci. USA.

[27] Ashburner M., Ball C.A., Blake J.A., Botstein D., Butler H., Cherry J.M., Davis A.P., Dolinski K., Dwight S.S., Eppig J.T. (2000). Gene ontology: Tool for the unification of biology. Nat. Genet..

[28] Gene Ontology Consortium (2019). The Gene Ontology Resource: 20 years and still GOing strong. Nucleic Acids Res..

[29] Schindelin J., Arganda-Carreras I., Frise E., Kaynig V., Longair M., Pietzsch T., Preibisch S., Rueden C., Saalfeld S., Schmid B. (2012). Fiji: An open-source platform for biological-image analysis. Nat. Methods.

[30] Pearson K. (1896). VII. Mathematical contributions to the theory of evolution.—III. Regression, heredity, and panmixia. Philos. Trans. R. Soc. Lond. Ser. AContain. Pap. A Math. Or Phys. Character.

[31] Narayanan P.K., Goodwin E.H., Lehnert B.E. (1997). α particles initiate biological production of superoxide anions and hydrogen peroxide in human cells. Cancer Res..

[32] Sue G.R., Ho Z.C., Kim K. (2005). Peroxiredoxins: A historical overview and speculative preview of novel mechanisms and emerging concepts in cell signaling. Free Radic. Biol. Med..

[33] Nicolussi A., D’Inzeo S., Capalbo C., Giannini G., Coppa A. (2017). The role of peroxiredoxins in cancer. Mol. Clin. Oncol..

[34] Thomas D.D., Ridnour L.A., Isenberg J.S., Flores-Santana W., Switzer C.H., Donzelli S., Hussain P., Vecoli C., Paolocci N., Ambs S. (2008). The chemical biology of nitric oxide: Implications in cellular signaling. Free Radic. Biol. Med..

[35] Pou S., Pou W.S., Bredt D.S., Snyder S.H., Rosen G.M. (1992). Generation of superoxide by purified brain nitric oxide synthase. J. Biol. Chem..

[36] Alderton W.K., Cooper C.E., Knowles R.G. (2001). Nitric oxide synthases: Structure, function and inhibition. Biochem. J..

[37] Xia Y. (2007). Superoxide Generation from Nitric Oxide Synthases. Antioxid. Redox Signal..

[38] Ignarro L.J., Buga G.M., Wei L.H., Bauer P.M., Wu G., Del Soldato P. (2001). Role of the arginine-nitric oxide pathway in the regulation of vascular smooth muscle cell proliferation. Proc. Natl. Acad. Sci. USA.

[39] Feng C. (2012). Mechanism of nitric oxide synthase regulation: Electron transfer and interdomain interactions. Coord. Chem. Rev..

[40] Villalobo A. (2006). Nitric oxide and cell proliferation. FEBS J..

[41] Ishii M., Shimizu S., Wajima T., Hagiwara T., Negoro T., Miyazaki A., Tobe T., Kiuchi Y. (2005). Reduction of GTP cyclohydrolase I feedback regulating protein expression by hydrogen peroxide in vascular endothelial cells. J. Pharm. Sci..

[42] Kim H.-L., Park Y.-S. (2010). Maintenance of cellular tetrahydrobiopterin homeostasis. BMB Rep..

[43] Karu T., Andreichuk T., Ryabykh T. (1993). Changes in oxidative metabolism of murine spleen following laser and superluminous diode (660–950 nm) irradiation: Effects of cellular composition and radiation parameters. Lasers Surg. Med..

[44] Vasquez-Vivar J., Martasek P., Whitsett J., Joseph J., Kalyanaraman B. (2002). The ratio between tetrahydrobiopterin and oxidized tetrahydrobiopterin analogues controls superoxide release from endothelial nitric oxide synthase: An EPR spin trapping study. Biochem. J..

[45] Kar S., Kavdia M. (2011). Modeling of biopterin-dependent pathways of eNOS for nitric oxide and superoxide production. Free Radic. Biol. Med..

[46] Dreyer J., Schleicher M., Tappe A., Schilling K., Kuner T., Kusumawidijaja G., Müller-Esterl W., Oess S., Kuner R. (2004). Nitric oxide synthase (NOS)-interacting protein interacts with neuronal NOS and regulates its distribution and activity. J. Neurosci..

[47] Ciesielska S., Bil P., Gajda K., Poterala-Hejmo A., Hudy D., Rzeszowska-Wolny J. (2019). Cell type-specific differences in redox regulation and proliferation after low UVA doses. PLoS ONE.

[48] Edgar R.S., Green E.W., Zhao Y., Van Ooijen G., Olmedo M., Qin X., Xu Y., Pan M., Valekunja U.K., Feeney K.A. (2012). Peroxiredoxins are conserved markers of circadian rhythms. Nature.

[49] Hoyle N.P., O’Neill J.S. (2015). Oxidation-reduction cycles of peroxiredoxin proteins and nontranscriptional aspects of timekeeping. Biochemistry.

[50] Powis G., Montfort W.R. (2001). Properties and biological activities of thioredoxins. Annu. Rev. Biophys. Biomol. Struct..

[51] Raffel J., Bhattacharyya A.K., Gallegos A., Cui H., Einspahr J.G., Alberts D.S., Powis G. (2003). Increased expression of thioredoxin-1 in human colorectal cancer is associated with decreased patient survival. J. Lab. Clin. Med..

[52] Grogan T.M., Fenoglio-Prieser C., Zeheb R., Bellamy W., Frutiger Y., Vela E., Stemmerman G., Macdonald J., Richter L., Gallegos A. (2000). Thioredoxin, a putative oncogene product, is overexpressed in gastric carcinoma and associated with increased proliferation and increased cell survival. Hum. Pathol..

[53] Hoshi Y., Tanooka H., Miyazaki K., Wakasugi H. (1997). Induction of thioredoxin in human lymphocytes with low-dose ionizing radiation. Biochim. Biophys. Acta Mol. Cell Res..

[54] Kakolyris S., Souglakos J., Georgoulias V., Giatromanolaki A., Sivridis E., Koukourakis M., Powis G., Gatter K.C., Harris A.L. (2001). Thioredoxin expression is associated with lymph node status and prognosis in early operable non-small cell lung cancer. Clin. Cancer Res..

[55] Morales Y., Nitzel D.V., Price O.M., Gui S., Li J., Qu J., Hevel J.M. (2015). Redox control of protein arginine methyltransferase 1 (PRMT1) activity. J. Biol. Chem..

[56] Napoli C., Paolisso G., Casamassimi A., Al-Omran M., Barbieri M., Sommese L., Infante T., Ignarro L.J. (2013). Effects of nitric oxide on cell proliferation: Novel insights. J. Am. Coll. Cardiol..

